# Fault Detection of a Roller-Bearing System through the EMD of a Wavelet Denoised Signal

**DOI:** 10.3390/s140815022

**Published:** 2014-08-14

**Authors:** Jong-Hyo Ahn, Dae-Ho Kwak, Bong-Hwan Koh

**Affiliations:** Department of Mechanical, Robotics and Energy Engineering, Dongguk University-Seoul, 30 Pildong-ro 1-gil, Jung-gu, Seoul 100-715, Korea; E-Mails: dkswhd83@gmail.com (J.-H.A.); daeho860@hanmail.net (D.-H.K.)

**Keywords:** fault detection, wavelet de-noising, empirical mode decomposition, intrinsic mode function, proper orthogonal value

## Abstract

This paper investigates fault detection of a roller bearing system using a wavelet denoising scheme and proper orthogonal value (POV) of an intrinsic mode function (IMF) covariance matrix. The IMF of the bearing vibration signal is obtained through empirical mode decomposition (EMD). The signal screening process in the wavelet domain eliminates noise-corrupted portions that may lead to inaccurate prognosis of bearing conditions. We segmented the denoised bearing signal into several intervals, and decomposed each of them into IMFs. The first IMF of each segment is collected to become a covariance matrix for calculating the POV. We show that covariance matrices from healthy and damaged bearings exhibit different POV profiles, which can be a damage-sensitive feature. We also illustrate the conventional approach of feature extraction, of observing the kurtosis value of the measured signal, to compare the functionality of the proposed technique. The study demonstrates the feasibility of wavelet-based de-noising, and shows through laboratory experiments that tracking the proper orthogonal values of the covariance matrix of the IMF can be an effective and reliable measure for monitoring bearing fault.

## 1. Introduction

Rotating machines are major components of energy transformation in power plant facilities. It is of primary importance for the rotating machines to be constantly and reliably operative if power production is to be organized cost-effectively. Increasing demands for preventive maintenance of rotating machinery have brought about enormous developments in fault monitoring techniques. The challenge of monitoring the condition of rotating machines is how to identify the component with a defect in the area where an increased vibration level has been noticed or measured, but simple measurement of the vibration signal is not a suitable means of detecting the damage and identifying its nature. Most of the methods exploit advanced signal processing techniques on a real-time basis, to separate fault-induced features from the vibration response of normal operating condition. Determining the adequate level of deviation from the baseline or healthy condition becomes a crucial portion of the fault detection process. Basically, successful condition monitoring consists of knowing what to listen to, how to interpret it, and when to provide timely maintenance.

A mechanical system having rotating components such as bearings and/or gears, provides a good example of condition monitoring. Specifically, bearing systems experience overload, misalignment, fatigue, looseness, and contamination, which can become major causes of cracks or spalls on the surface of the inner or outer-race. Typically, fault-induced signals from rotating machinery involve periodical impulses that are masked by environmental noises, along with the high frequency dynamics of structural components of rotating components [1]. The spectral signatures of good and defective bearings have been ascertained, and widely explored in a variety of literatures. Craig *et al.* introduced a condition monitoring technique using electrostatic charge. The study uses electrostatic wear site sensing to identify unexpected failure of the support bearing [2]. Sugumaran and Ramachandran employed a decision tree to automatically extract statistical features, and different fault conditions of the roller bearing are classified. Also, a fuzzy classifier is developed and tested with representative data, using the decision tree [3]. Zhang *et al.* applied a genetic programming-based classification approach for condition monitoring of a bearing system. They showed that the selection of damage-sensitive features using genetic programming outperformed other methods, such as artificial neural network and support vector machine in conjunction with genetic algorithms [4]. In regard to wavelet-based approaches, Ericsson *et al.* compared several different vibration analysis techniques to detect the local faults in a bearing. The study concluded that wavelet-based methods are particularly well suited to monitoring the bearing system [5]. Also, Widodo and Yang used wavelet support vector machine for classifying fault in an induction motor, during the transient start-up stage. They employed five decomposition levels, to extract the difference caused by faults in some frequency ranges [6]. Jung and Koh [7] applied a wavelet-based vertical energy threshold technique to locate damages in a truss structure. The study showed that silhouette statistics could be effectively used to assess the quality of clustering of damage-sensitive features.

The most critical portions of developing a successful condition monitoring technique are signal processing [8], feature extraction, and the interpretation of information [9]. For signal processing, wavelet-based de-noising and AR-based filter techniques have been widely introduced in recent years. Vijay *et al.* evaluated wavelet-based de-noising schemes for bearing condition classification [10]. Junsheng, Dejie and Yu proposed an AR-based fault detection method for roller bearing in conjunction with EMD [11]. Dharap, Koh and Nagarajaiah investigated ARMarkov observer, to determine the severity of damage, and monitor the progress of damage propagation in truss structure. Unlike Kalman filter-based methods, ARMarkov observer does not require noise statistics, or initial conditions of the system [12].

The nonlinear and non-stationary nature of a bearing signal inevitably also demands time-frequency analyses, such as the Hilbert-Huang transform (HHT) [13]. In particular, the empirical mode decomposition (EMD) process of HHT breaks a non-linear and non-stationary signal into a finite number of modes or intrinsic mode functions (IMF) [14]. In regard to the feature extraction, proper orthogonal decomposition (POD) has recently gained much attention, for its benefit in rescaling empirical components from a high-dimensional data set [15,16]. The underlying theory of POD is closely related to principal component analysis (PCA) and singular value decomposition (SVD).

In order to characterize the presence of defect, peak-sensitive statistical indicators, such as root-mean-square (RMS), kurtosis [17], and crest factor, have been widely explored for bearing condition monitoring. The crest factor is calculated from the peak amplitude of the waveform divided by the RMS value of the waveform. In regard to RMS and crest factor, some studies report their limitation in detecting localized defects [18]. The theoretical motivation of using kurtosis is that statistical moments of the data, *i.e.*, the fourth moment, which is normalized with respect to the fourth power of standard deviation, can be a good indicator for bearing fault. A bearing in good condition produces the probability density of acceleration having Gaussian distribution; the kurtosis value approaches 3. Thus, the kurtosis value above 3 can be a threshold for indication of fault in a bearing. However, for some cases, such as fully advanced damages in bearing, kurtosis could not successfully detect the defect [19].

Although an enormous amount of literature has been published in regard to condition monitoring techniques, it is still a challenging task to find and implement a reliable method for real-world problems. We investigate the fault detection problem of a roller bearing system, through monitoring the variation of proper orthogonal values of the covariance matrix developed from the highest frequency component, *i.e.*, the IMF of signals decomposed by EMD. All the measured bearing vibration signals are de-noised through a wavelet-based thresholding function, before carrying out fault detection. For comparison, we also present the kurtosis value of a fault signal, to substantiate the feasibility of an IMF-based fault detection approach.

## 2. Signal Processing and Feature Extraction

### 2.1. Wavelet-Based De-Noising

Recently, wavelet transforms and related signal processing algorithms have been widely investigated for potential application to the condition monitoring of rotating machinery [9,20]. Unlike a bandwidth-based low-pass filter, the wavelet de-noising scheme does not influence the fundamental nature of the signal, because the wavelet transform may remove the noise, according to simultaneous rescaling in both the frequency and time domain. Noise corrupted vibration signal, especially with sharp transients, can be de-noised through the thresholding function in wavelet domains [21,22]. Depending on properly selected threshold rules, detail coefficients of the decomposed signal are selectively preserved or suppressed. Consider that an original (noise-free) signal *v*(*k*) is being corrupted by unwanted noise *g*(*k*), with unknown noise level statistics σ. Hence, the obvious goal of de-noising is to faithfully recover *v*(*k*), without jeopardizing its inherent nature, from observation *y*(*k*):

(1) y(k)=v(k)+σg(k)

The successive steps of wavelet-based de-noising are as follows: firstly, carry out wavelet transform of the targeted noisy signal for *N* levels. Secondly, select the appropriate thresholding value (μ), to eliminate unwanted noise up to *N* levels. Finally, the de-noised signal may be reconstructed through inverse wavelet transform of the modified detail coefficients. The thresholding rule decides whether the coefficient that constitutes the original signal is to be retained or eliminated. Donoho and Johnstone suggest two types of thresholdings [22], *i.e.*, hard and soft thresholding: the hard thresholding (Equation (2)) puts all the signal values smaller than μ to zeros, while the soft thresholding rule (Equation (3)) additionally retains the differences between μ and the signal value larger than μ. Here, *w*(*x*) is a thresholding function:

(2) $$w\left(x\right)=\begin{array}{c} \;x,\;\left|x\right|\geq \mu \\ \;0,\;\left|x\right|<\mu \end{array}$$

(3) $$w\left(x\right)=\{\begin{array}{c} \text{sign}\left(x\right)\left(\left|x\right|-\mu \right),\;\left|x\right|\geq \mu \\ \;0,\;\left|x\right|<\mu \end{array}$$

### 2.2. Empirical Mode Decomposition *(*EMD*)*

Recently, a generalized form of spectral analysis, the Hilbert-Huang transform (HHT), has been developed to tackle non-stationary and nonlinear signal. HHT-based signal processing has two components: the Hilbert spectral transform [13], and empirical mode decomposition (EMD). The major limitations of Fourier spectral analysis are the assumptions of stationarity and linearity of the underlying signal. It is known that the Hilbert transform of an arbitrary time series *x*(*t*) is defined as:

(4) $$Y\left(\text{t}\right)=\frac{1}{\pi }\int_{-\infty }^{\infty }\frac{x\left(\tau \right)}{t-\tau }d\tau$$

Note that Equation (4) is equivalent to the convolution product of *x*(*t*) and 1/*t*. The Hilbert transform can be used to calculate instantaneous frequencies and amplitudes, to describe the local nature of a signal, such as energy of frequency and time. On the other hand, EMD decomposes almost any form of signals into a finite set or function having instantaneous frequency value. The outcome of EMD is known for the intrinsic mode function (IMF). Basically, EMD belongs to a signal processing technique that breaks a temporal signal into empirically characterized modes, as the EMD decomposes the original signal into several IMFs, by subsequently removing the lower frequency components from the highest frequency of the residual of its ancestor, which is also known as the sifting process.

The process of EMD is as follows: first, all the local extrema and minima of *x*(*t*) should be identified, and connected by cubic spline line to form upper and lower envelopes of the signal. Then, the original signal is subtracted by the mean value (*m*_1_ of the upper and lower envelopes, *i.e.*, *x*(*t*) − *m*_1_ = *h*_1_, which becomes ideally the first IMF (*h*_1_). If *h*_1_ is not an IMF, *h*_1_ is treated as the original signal, and the previous steps are repeated; then *h*_1_ − *m*_11_ = *h*_11_. The iterative decomposition process of enveloping and subtracting toward residual value from the previous IMF is called shifting. After repeated shifting up to *k* times, *h*_1_ becomes an IMF, that is *h*_1(_*_k_*_−1)_ − *m*_1_*_k_* = *h*_1_*_k_*. It is also denoted that *c*_1_ = *h*_1_*_k_*. Separating *c*_1_ from *x*(*t*) through *r*_1_ = *x*(*t*) − *c*_1_, the procedure is repeated up to *n* times, until the *r_n_* becomes a monotonic function *r_q_*, where no more IMFs can be extracted. While the first extracted IMF retains interpolation of local maxima, the last IMF, conveniently called the residual, represents the lowest frequency component, or simply the trend of the signal. Having being fully decomposed, the original signal *x*(*t*) can be perfectly reconstructed through summing up all the IMFs, including the final residue *r_q_*, as shown in Equation (5):

(5) $$x\left(t\right)=\sum_{i=1}^{q}c_{i}+r_{q}$$

Note that a signal has to satisfy two assumptions to be eligible for EMD: (1) the difference between the number of zero-crossings and the number of extrema should be less than one for the whole data set, and (2) the mean value of the upper envelop and lower envelop should be zero at any point. Again, the observed signal for the EMD doesn't have to be linear or stationary. Knowing that the signal from rotating machinery exhibits a similar nature of non-linear and non-stationary characteristics, many researchers have investigated HHT for diagnosing damage in rotating machinery [23–25].

### 2.3. Proper Orthogonal Decomposition (POD)

Modeling and verification of a dynamic system demands a statistical technique that projects a dominant mode to its subspace, namely proper orthogonal decomposition (POD), which is also known as Karhunen-Loève decomposition [26]. The underlying nature of POD represents the empirical modes of a system, and is identical to singular value decomposition (SVD), or principal component analysis (PCA) [15,16]. Referring to Kerschen and Golinval [15], the fundamental goal of POD is to find the basis function σ(*x*) of the following extreme value in a continuous function of *x* in Ω:

(6) $$\text{Maximize}\left\{\lambda =\frac{(1/N)\sum_{i=1}^{N}{\left(\int_{\Omega }\sigma (x)\sigma (x)d\Omega \right)}^{2}}{\int_{\Omega }\sigma (x)\sigma (x)d\Omega }\right\}$$

The solution of the maximization problem of Equation (6) can be reduced to the following integral eigenvalue problem:

(7) $$\int_{\Omega }R(x,x^{'})\sigma (x^{'})dx^{'}=\lambda \sigma \left(x^{'}\right)$$

Here, *R*(*x*, *x*′) is an averaged auto-correlation function. Equation (7) yields orthogonal eigenfunctions and eigenvalues, or equivalently proper orthogonal modes (POM) and corresponding proper orthogonal values (POV). In practice, given an *m* × *n* matrix, *i.e.*, *m* observations of *y*(*t*), sampled *n* times, *Y* is shown as below:

(8) $$Y=\left[\begin{array}{ccc} y_{11} & \ldots & y_{1n}\\ \vdots & \cdots & \vdots \\ y_{m1} & \ldots & y_{mn} \end{array}\right]$$

The proper orthogonal value (POV) is equivalent to the singular value of the covariance matrix *A*, which is an ensemble of snapshots or observations from sensors on the bearing system [27,28]:

(9) $$A=\left(\frac{1}{n}\right)YY^{T}$$

### 2.4. POV of IMF Matrix

This study exploits the correlation of POV of the IMF matrix and the bearing fault through the matrix *A* of collected vibration measurement from a bearing system. It is also well known that the kurtosis value of measured signals can be an indicator for the presence of bearing fault. The kurtosis is normally defined as the fourth population moment, normalized with respect to the fourth power of standard deviation, or Equation (10), where *E* is the expectation operator, and μ is the mean:

(10) $$\beta =\frac{E{\left(X-\mu \right)}^{2}}{{\left(E{\left(X-\mu \right)}^{2}\right)}^{2}}$$

**Figure 1.**
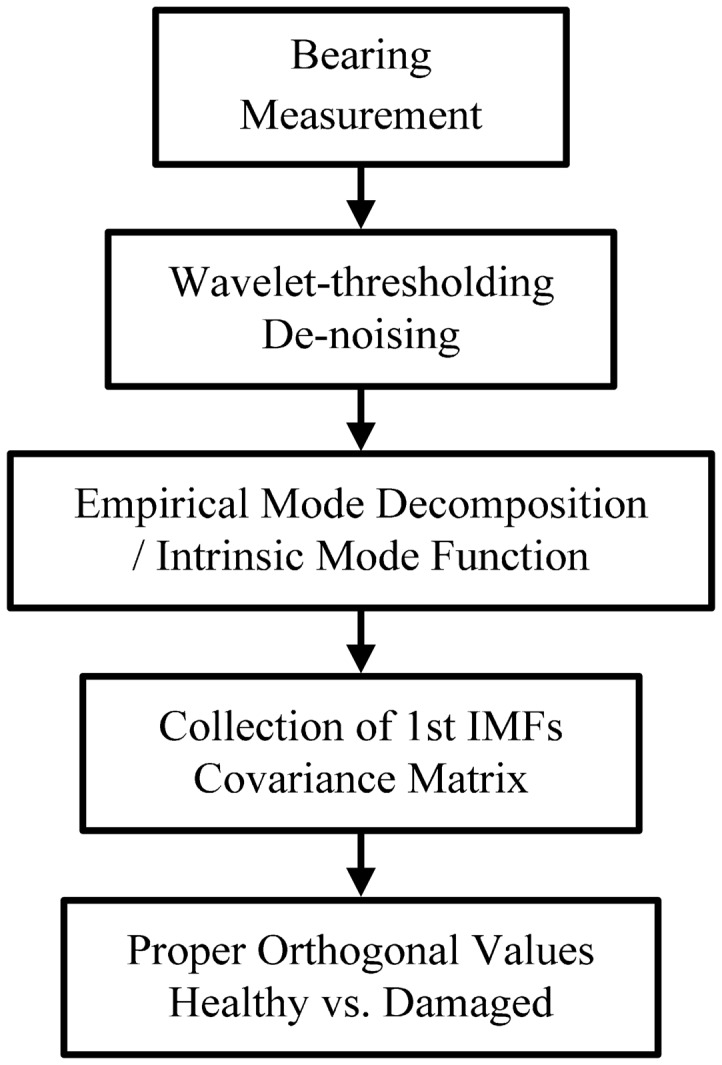
Flow chart of the roller bearing fault monitoring method.

Here, we compare the condition monitoring results of a roller-bearing system, using the change of kurtosis value, and the POVs of an IMF covariance matrix. Figure 1 illustrates a flow chart of the roller bearing fault monitoring method. This describes the overall process from the original signal to the filtered one, and the consequent decomposition steps for calculating POVs.

## 3. Experimental Setup and Data Collection

**Figure 2.**
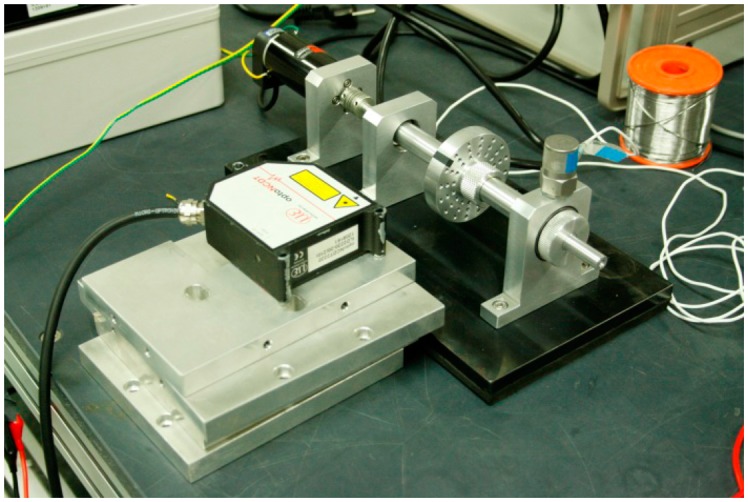
A roller bearing system with driving motor and acceleration sensor instrumentation.

**Figure 3.**
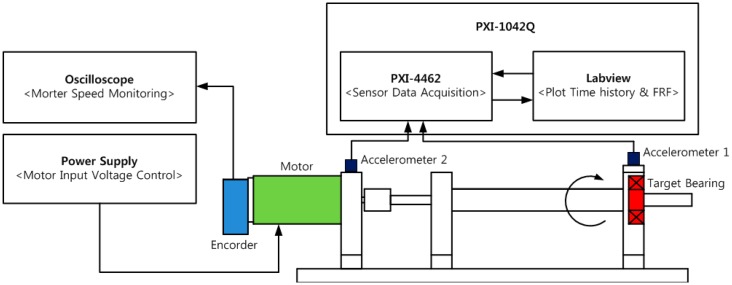
Schematic drawing of the experimental setup for the roller bearing system.

**Figure 4.**
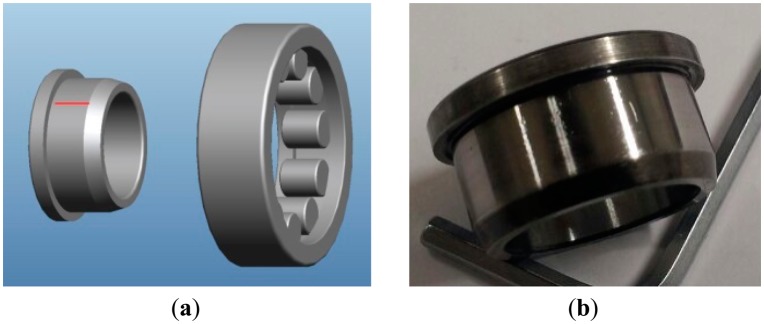
(**a**) Inner/outer race of roller bearing; (**b**) Scratch-type defect on the inner race.

To develop and validate a fault detection technique for a rotating system, it is important to design the apparatus to facilitate the frequent replacement of bearing modules for damaged and healthy conditions of the system, without disturbing the boundary conditions. Also, the dynamic signature of the defect in the component should be transmitted to the measurement point. Figures 2 and 3 show the experimental setup for monitoring the vibration signal of a roller bearing system, and its schematic drawing. As shown in the figures, the driving motor and the main shaft are connected through mechanical coupling. The speed of the shaft is measured through an optical encoder, while a 500 mv/g accelerometer of PCB is mounted on the vertical direction of the bearing housing for vibration measurement. The data acquisition (DAQ) system, NI PXI-1042Q collects the bearing condition signal with a sampling speed of 50 KHz, through a LabVIEW interface. The roller-bearing type in this experiment is NJ 202 ECP from SKF, withstanding static loads up to 12.5 KN, at a rotating speed of 22,000 rpm. Note that a scratch-type bearing fault is imposed on the surface of the inner race, as shown in Figure 4. First, the DAQ collects a set of vibration measurements of the healthy bearing, and another set of measurement is recorded, after having imposed the defect on the healthy bearing.

**Figure 5.**
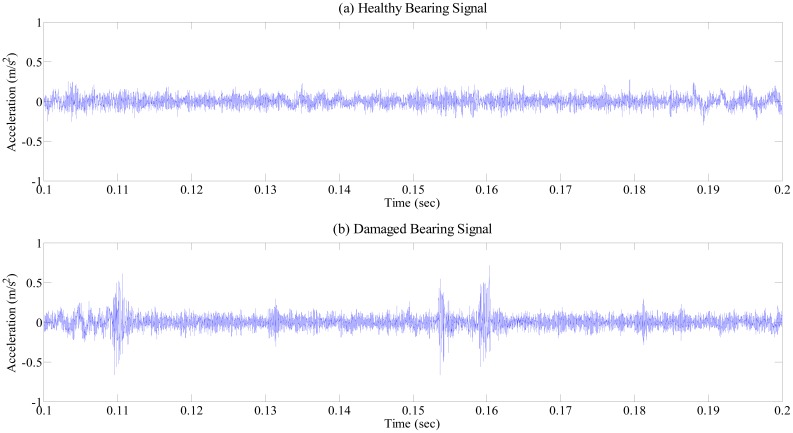
Measured bearing signals: (**a**) healthy condition; and (**b**) damaged condition.

Figure 5 shows the time history data measured from the bearing system between 0.1 and 0.2 s without de-noising: one without damage (Figure 5a) and the other one (Figure 5b) having the scratch damage of Figure 4. Figure 5 clearly exhibits the repeated peaks caused by the periodic impact between the faults in the race surface and rolling elements. Obviously, high frequency components are identified between the two repeated peaks that are considered to be an uncorrelated combination of resonance reflection and measurement noise. Apparently, wavelet-based de-noising capability enhances the signal-to-noise ratio of signals, by removing noise, without affecting high-peak stress waves caused by bearing damage.

## 4. Fault Detection and Discussions

**Figure 6.**
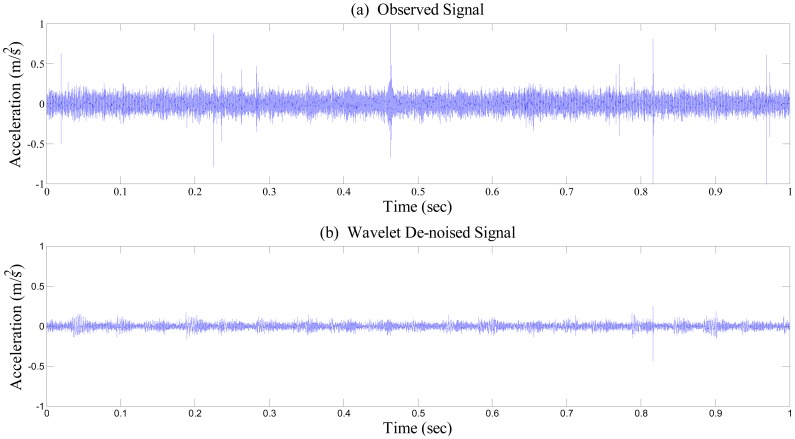
Time-history data of healthy bearing signal (**a**), and wavelet-based de-noised signal (**b**).

**Figure 7.**
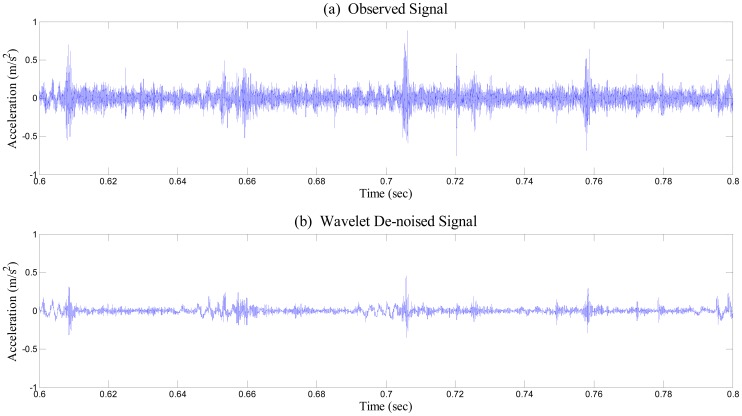
Time-history data of damaged bearing signal (**a**), and wavelet-based de-noised signal (**b**).

Again, Figures 6 and 7 illustrate before and after performing wavelet de-noising towards the time series of bearing vibration. As shown in Figure 6a, irregular spikes caused by measurement noise are inconsistently positioned in the vibration signal, which may yield extra harmonics to the frequency response. Because the relative level of deviation from the baseline or healthy state of the bearing system is a crucial factor for feature extraction before deciding the presence of fault in the system, the signal-to-noise ratio has to be enhanced as much as possible. Although numerous schemes for noise removal exist, conventional low-pass type filters are inadequate for monitoring a bearing system. The impulse caused by collisions between faults and the rotating component excites the entire bearing system. Thus, the resulting impulse response contains high-frequency content, which becomes a critical evidence of damage presence, as shown in Figure 7.

**Figure 8.**
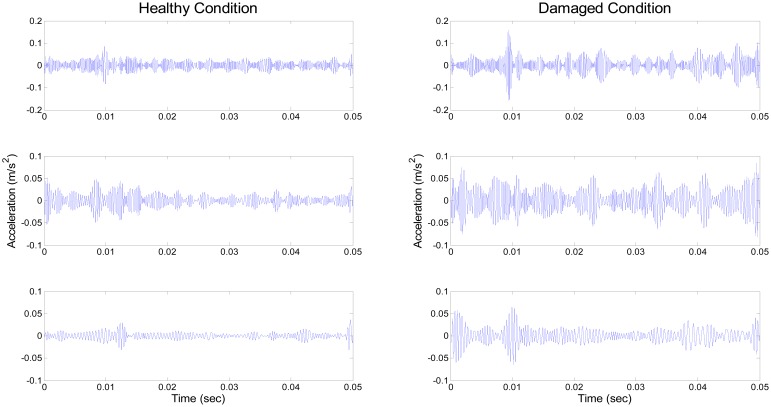
The first three IMFs of healthy (**left**), and damaged bearing (**right**) signals.

Apparently, wavelet thresholding has successfully removed fault-irrelevant low-amplitude high-frequency wave components. Here, the study incorporates de-noising through the wavelet coefficient thresholding principle of soft thresholding without rescaling. Having been de-noised, elastic waves due to collision of the defect remain intact, as shown in Figure 7b. Although the overall power of the signal has been moderately reduced, the repeated peaks clearly remain after the wavelet de-noising process. After de-noising, we equally segmented both the measured healthy and damaged signals into 10 segments. Using these de-noised segments, we carried out EMD, to extract IMFs for both healthy and damaged bearing signal segments (see Figure 8).

**Figure 9.**
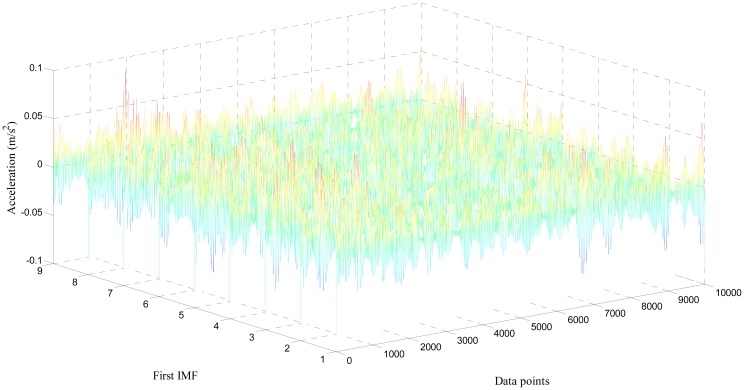
Profile of the collection of the first IMFs from healthy bearing acceleration: 10 segments.

**Figure 10.**
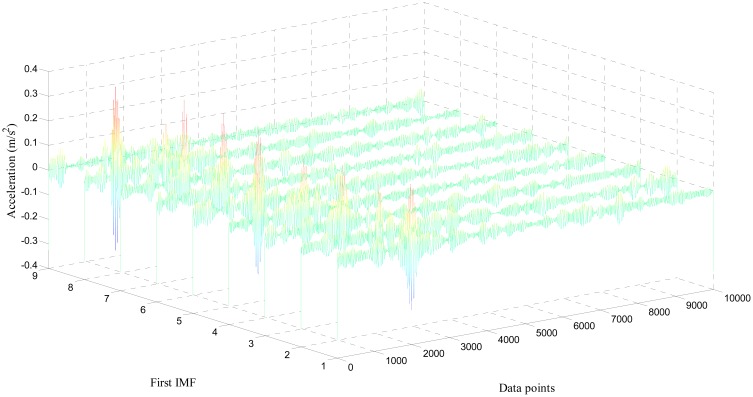
Profile of the collection of first IMFs from damaged bearing acceleration: 10 segments.

Only the first three IMFs produced by EMD for both healthy and damaged bearing signal are depicted in Figure 8, due to space constraints. Because higher intrinsic modes mostly incorporate high-amplitude peaks, resulting from repeated collisions between the fault spot and moving elements of the bearing system, we used the first three IMFs to develop the covariance matrix [29,30]. As shown in Figure 7, IMFs from the damaged condition exhibit a noticeable number of peaks, and relatively stronger power of signals, compared to the healthy ones. This study collected the first IMFs of a total of nine sets of both healthy and damaged bearing data (each set has 10,000 data points). Figures 9 and 10 visualize the waterfall profile of amplitude *vs.* data points of stacked IMFs extracted from healthy and damaged bearing measurements, respectively. As shown in Figure 10, the collection of the first IMFs from the data sets of the damaged case shows prominent high-amplitude peaks. In contrast, the IMF collection of the healthy case doesn't provide any significant peak or fluctuation, and so is visually similar to white noise (see Figure 9). The prominent difference of the first IMFs between healthy and damaged bearing signals becomes the damage-indicative feature for extracting POVs of its covariance matrix.

**Figure 11.**
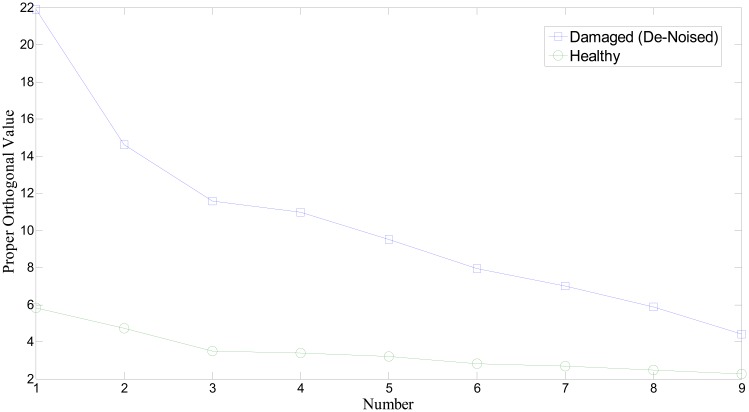
Proper orthogonal values of the collected covariance matrix of the IMFs from segments.

**Figure 12.**
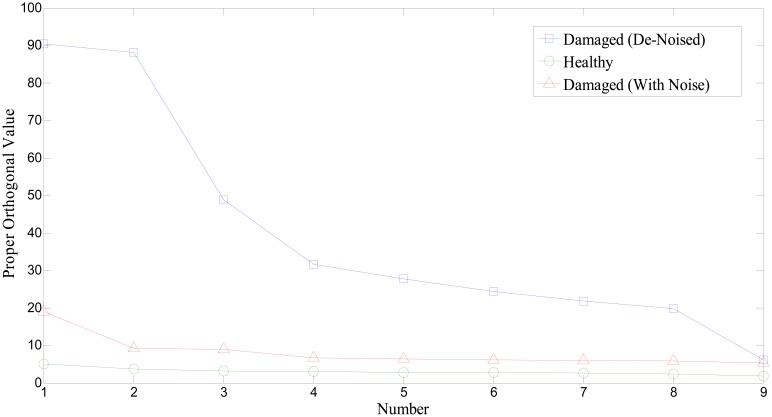
Proper orthogonal values of the collected covariance matrix of the IMFs from noised and wavelet-based de-noised segments (additional test set).

Figure 11 shows the profile of singular values of the covariance matrix of the collection of the first IMFs. It is obvious that singular values from the fault bearing vibration signal provide higher than normal bearing. Note that we used an additional bearing signal set to improve the reliability and verification of the aforementioned approach. Figure 12 illustrates a comparison of singular values on a different set of bearing signals. Again, the singular value of the covariance matrix is equivalent to the proper orthogonal value of the ensemble of responses. The absolute level of POVs and their ordering pattern apparently differentiate the state or condition of the bearing system. In particular, wavelet-based de-noising plays a significant role in enhancing the detectability of bearing defect (see Figure 12). Conclusively, a different level of orthogonality caused by prominent peaks of the first IMF in the damaged bearing signal is a critical feature for the presence of fault. Thus, in this study, we propose that the set of highest frequency components from EMD and its singular values can be used as a potential damage-sensitive feature, to monitor the condition of the bearing system. Note that the number of segments to form the covariance matrix limits the number of POVs. This study found that around 10 to 15 segments would be enough to produce a clear deviation between healthy and damaged bearing conditions.

**Figure 13.**
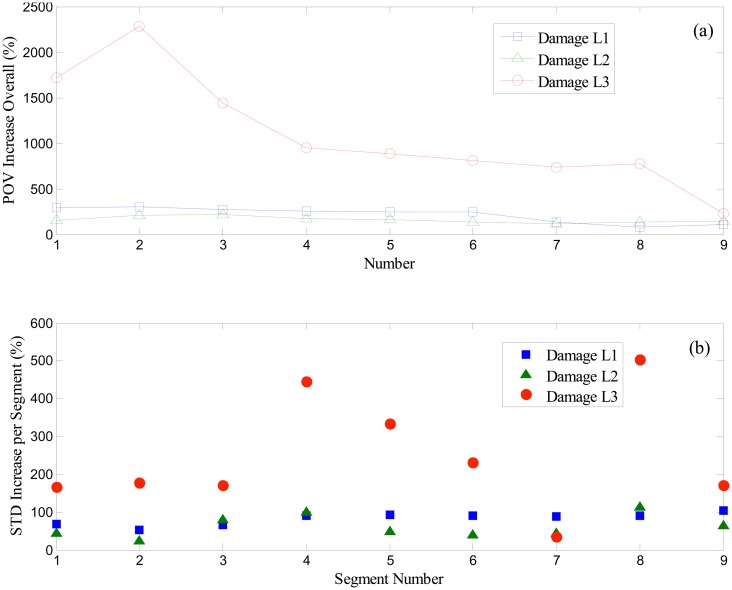
Comparison of increasing ratio between (**a**) the proper orthogonal value of total segments, and (**b**) standard deviation of the first IMF from the wavelet-based de-noised segment (*L1* & *L2*: moderate damage, *L3*: severe damage).

Figure 13 compares the increasing ratio (in percentile) of POV of the first IMFs of all segments, and the standard deviation of the first IMF of each segment, *i.e.*, before and after the occurrence of a scratch-type defect in roller bearing. Here, damage *L1 ∼ L3* cases are separate sets of experimental data acquired from three different damage levels, where cases *L1* and *L2* represent moderate damage severity. The most severe level of damage is denoted as case *L3*. As shown in Figure 13a,b, as the intensity of damage deepens, both POV of total segments and the standard deviation of each segment increase. However, in all three cases the overall level of POV varies much larger than the standard deviation. While increasing ratios of standard deviation of *L1* and *L2* are approximately positioned in the 100% band for each segment, POVs of the same case increase up to 300%, compared to the healthy bearing. In the case of *L3*, there exists a significant gap of increasing ratio between the POV and the standard deviation. Also, note that the segment number 7 of case *L3* provides a contrasting result of standard deviation, as shown in Figure 13b.

**Figure 14.**
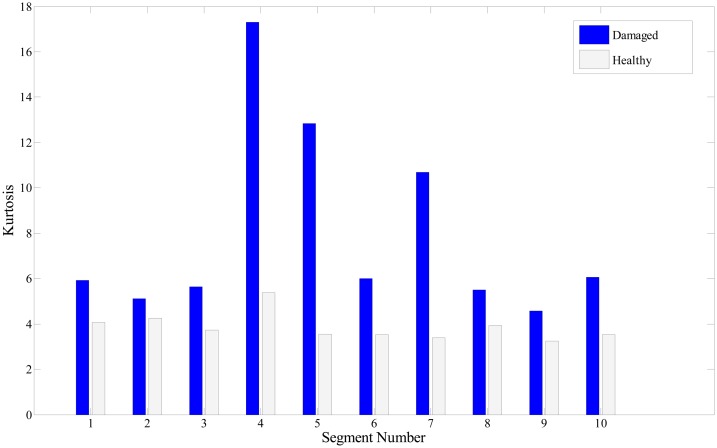
Kurtosis value of 10 segments.

**Figure 15.**
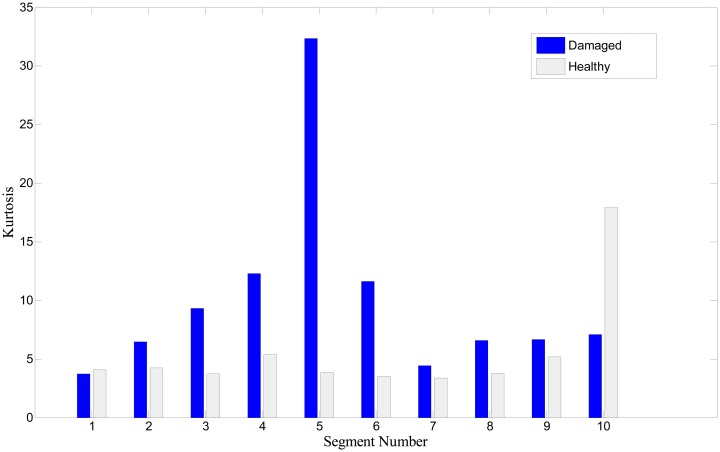
Kurtosis value of 10 segments (additional test set).

In this study, the kurtosis indicator for quantifying the presence of a peak and its intensity is applied to the bearing signals, for comparison. Figure 14 shows the kurtosis values of 10 segments or data sets from both healthy and damaged roller bearing signals. Obviously, a signal segment from a damaged bearing takes higher kurtosis values, than from a healthy one. If background Gaussian noise masks the signal of interest, however, kurtosis may easily fail to indicate the presence of fault. This point is clearly described in Figure 15, where the 10th segment exhibits a higher value of kurtosis for the healthy case.

In summary, the same set of signals is used for two methods: one is inspecting the POV of the IMF covariance matrix, and the other is checking the kurtosis value. Although we see the gap of kurtosis value from fault signal over the healthy one, some segments, *i.e.*, No. 2 of Figure 14, and No. 1, 7, and 10 of Figure 15, show insufficient or misleading diagnosis for the bearing condition monitoring. Performing POV of the IMF covariance matrix on the bearing signal is likely to improve the quality of the diagnosis results. Results show that the first POV from the fault signal is 20 times higher than that from the healthy signal. This result suggests that observing the variation of the POV of the IMF provides insight into the nature of abnormality in a signal, and can be further developed as a reliable method for roller bearing diagnosis.

## 5. Conclusions

In this paper, we perform an experimental validation of a condition monitoring system for a roller-bearing system. Here, we assumed the bearing fault was a scratch-mark on the surface of the inner race. Before performing fault detection, we carried out wavelet-based denoising through a soft-thresholding scheme. Unlike the conventional low-pass filter, the wavelet-based denoising method does not distort the elastic waves due to collision of the defect in bearing. After denoising, we carried out EMD to extract the IMFs of collected segments of vibration signals. Finally, we compared the POV of collected IMFs before and after the occurrence of bearing defects. The present method provides a simple and intuitive approach to exploit damage-sensitive features, namely the POV from the IMF matrix of the bearing signals. Although the kurtosis value could be used to characterize repeating peaks due to faulty bearing, the proposed method of using the POV produced more reliable fault detection results in some cases. We will extend this study in the near future to more challenging cases, in which multiple sources of faults co-exit, such as a bearing dismantled due to fatigue, and cracks in a gear tooth of a gearbox system.
